# Role of metabolites of cyclophosphamide in cardiotoxicity

**DOI:** 10.1186/s13104-017-2726-2

**Published:** 2017-08-14

**Authors:** Koichiro Kurauchi, Takuro Nishikawa, Emiko Miyahara, Yasuhiro Okamoto, Yoshifumi Kawano

**Affiliations:** Department of Pediatrics, Graduate School of Medical and Dental Sciences, Kagoshima University, Sakuragaoka 8-35-1, Kagoshima City, Kagoshima Prefecture 890-8520 Japan

**Keywords:** Acrolein, Aldehyde dehydrogenase, Cardiotoxicity, Cyclophosphamide, *N*-acetylcysteine

## Abstract

**Background:**

The dose-limiting toxic effect of cyclophosphamide (CY) is cardiotoxicity. The pathogenesis of myocardial damage is poorly understood, and there is no established means of prevention. In previous studies, we suggested that for CY-induced cardiotoxicity, whereas acrolein is the key toxic metabolite, carboxyethylphosphoramide mustard (CEPM) is protective. We sought to verify that acrolein is the main cause of cardiotoxicity and to investigate whether aldehyde dehydrogenase (ALDH), which is associated with greater CEPM production, is involved in the protective effect for cardiotoxicity. We also evaluated the protective effect of *N*-acetylcysteine (NAC), an amino acid with antioxidant activity and a known acrolein scavenger.

**Methods:**

H9c2 cells were exposed to CY metabolites HCY (4-hydroxy-cyclophosphamide), acrolein or CEPM. The degree of cytotoxicity was evaluated by MTT assay, lactate dehydrogenase (LDH) release, and the production of reactive oxygen species (ROS). We also investigated how the myocardial cellular protective effects of CY metabolites were modified by NAC. To quantify acrolein levels, we measured the culture supernatants using high performance liquid chromatography. We measured ALDH activity after exposure to HCY or acrolein and the same with pre-treatment with NAC.

**Results:**

Exposure of H9c2 cells to CEPM did not cause cytotoxicity. Increased ROS levels and myocardial cytotoxicity, however, were induced by HCY and acrolein. In cell cultures, HCY was metabolized to acrolein. Less ALDH activity was observed after exposure to HCY or acrolein. Treatment with NAC reduced acrolein concentrations.

**Conclusions:**

Increased ROS generation and decreased ALDH activity confirmed that CY metabolites HCY and acrolein are strongly implicated in cardiotoxicity. By inhibiting ROS generation, increasing ALDH activity and decreasing the presence of acrolein, NAC has the potential to prevent CY-induced cardiotoxicity.

## Background

Hematopoietic stem cell transplantation (HSCT) is a radical but risky treatment for hematopoietic diseases. For HSCT, alkylating agent cyclophosphamide (CY) is one of the mainstay drugs, used in high doses during most conditioning regimens [1]. Recently, administration of posttransplantation CY (PTCy) in high doses has also been attracting attention as a novel strategy for preventing graft-versus-host disease (GVHD) [2, 3].

**Fig. 1:**
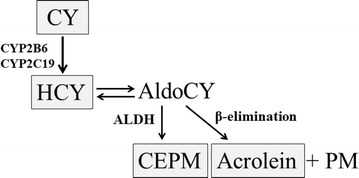
Cyclophosphamide metabolic pathways. Cyclophosphamide (CY) is metabolized to 4-hydroxy-cyclophosphamide (HCY) in the hepatic cytochrome P-450 enzyme (CYP) system (CYP2B6 and/or CYP2C19). HCY enters cells as tautomer aldocyclophosphamide (AldoCY). Through β-elimination, AldoCY can be converted to phosphoramide mustard (PM) and acrolein. Alternatively, AldoCY can also be oxidized to the inactive metabolite *o*-carboxyethylphosphoramide mustard (CEPM) by aldehyde dehydrogenase (ALDH)

CY is a prodrug activated by the hepatic cytochrome P-450 (CYP) enzyme system to produce 4-hydroxycyclophosphamide (HCY), which forms in equilibrium with aldocyclophosphamide (AldoCY). Depending on cell type, AldoCY may, through the chemical process of β-elimination, decompose to form cytotoxic phosphoramide mustard (PM) and byproduct acrolein, or may be mediated by aldehyde dehydrogenase (ALDH), to inactive metabolite *o*-carboxyethyl-phosphoramide mustard (CEPM) [4, 5] (Fig. 1).

As reported in several cases, the dose-limiting toxicity of CY is cardiotoxicity, which has been observed only after high-dose CY therapy [6–8]. Most severely, with attendant risk of sudden death, this may manifest as hemorrhagic necrotic perimyocarditis. On the basis of postmortem examination, the pathophysiology of high-dose CY-associated cardiac toxicity is thought to depend on toxic endothelial damage followed by extravasation of toxic metabolites, resulting in myocyte damage, interstitial hemorrhage, and edema [9, 10].

The pathogenesis of myocardial damage is poorly understood, and there is no established means of prevention. In our previous study [11], to evaluate myocardial cell injury by CY, we exposed rat cardiac myocardial cell line H9c2 to CY metabolized in the S9 fraction of rat liver homogenates. There was no evidence of myocardial cell injury from CY alone, such injury was only evident when CY was metabolized in vitro [11]. We speculated that the pathogenesis of myocardial cell injury was possibly due to an increase in acrolein and apoptosis. Subsequently, we found that co-exposure with *N*-acetylcysteine (NAC), an amino acid with antioxidant activity and a known acrolein scavenger [12], was able to suppress myocardial cell injury. It seemed that the mechanisms of NAC protection against CY-induced cardiotoxicity involved inhibition of apoptosis, acrolein suppression, and increased production of CEPM [11]. We consequently decided to investigate whether ALDH activity plays a role in lessening myocardial cell injury by increasing the production of CEPM.

We sought to convincingly demonstrate that acrolein is the main cause of CY-induced cardiotoxicity and to investigate how to cause the cytotoxic activity for H9c2 cardiomyocytes. We also evaluated the relationship between myocardial cell injury and ALDH activity.

## Methods

### Reagents

CY (Cat. #C0768), dimethyl sulfoxide (DMSO: Cat. #D2650), NAC (Cat. #A9165), and metaphosphoric acid (MPA: Cat. #239275) were purchased from SIGMA-Aldrich (St. Louis, MO), HCY (Cat. #sc-206885) and CEPM (Cat. #sc-207411) were purchased from Santa Cruz Biotechnology (Dallas, TX). Acrolein (Cat. #M-815B/5031-03) was purchased from AccuStandard (New Haven, CT). CY and NAC were diluted with normal saline. HCY and CEPM were diluted with phosphate-buffered saline (PBS) and DMSO, respectively.

### Cell culture

H9c2 cell line embryonic rat cardiomyocytes (Cat. #CRL-1446) were supplied by the American Type Culture Collection (ATCC, Manassas, VA). The H9c2 specimens were maintained in DMEM medium (Cat. #11995-065; Life Technologies, Carlsbad, CA) containing 10% fetal bovine serum (FBS: Cat. #30-2020; also from ATCC) at 37 °C in a humidified atmosphere with 5% carbon dioxide (CO_2_).

### Analysis of cell viability

H9c2 cells were plated at 2.5 × 10^4^ cells/mL density in 24-well plates and grown overnight. These samples were exposed to CY (250 μM), HCY (10 or 30 μM), CEPM (10 or 30 μM) or acrolein (10 or 30 μM) at 37 °C in 5% CO_2_ for 24 or 48 h. After the addition to each sample of 50 μL of 5 mg/mL of 3-(4,5-dimethyl-2-thiazolyl)-2,5-diphenyl-2*H*-tetrazolium bromide (MTT: Cat. #345-01821; Dojindo, Kumamoto, Japan), samples were incubated at 37 °C in 5% CO_2_ for 2 h. Formazan crystals were dissolved by adding 1 mL of DMSO and, using a microplate reader (Infinite M200, Tecan, Mannedorf, Switzerland), and this data was analyzed using i-Control Ver. 1.0 software (Tecan).

To investigate the influence of NAC, before exposure to CY or CY metabolites, H9c2 cells were incubated for 2 h with 10% FBS supplemented DMEM containing 1 mM NAC. For these protocols, we conservatively selected a known nontoxic concentration, 1 mM, of NAC [11]. After pre-treatment, the medium of each sample was replaced with fresh medium containing NAC along with CY or CY metabolites. In parallel protocols, the morphology of H9c2 cells after exposure, in the 24-well culture plates, to CY or CY metabolites was observed using 100× magnification fluorescence microscopy (Axio Observer Z1; Zeiss MicroImaging GmbH, Göttingen, Germany). For control, unexposed H9c2 cells were seeded in 24-well plates at 2.5 × 10^4^ cells/mL density in DMEM with 5% FBS and incubated at 37 °C in 5% CO_2_.

### Measurement of intracellular ROS generation

To detect intracellular ROS, we used dichlorofluorescin diacetate (DCFH-DA) molecular probes (Cat. #D6883; SIGMA-Aldrich) as our previous study [11]. Briefly, H9c2 cells were harvested and re-suspended in ice-cold PBS. And then, 5 mM DCFH-DA was added to the cell suspension. After 15 min of incubation at 37 °C in 5% CO_2,_ 10 or 30 mM HCY or 30 mM acrolein was added and incubated 37 °C in 5% CO_2_ for 15 min. The fluorescence intensity was evaluated using a flow cytometer (Accuri C6, Becton, Dickinson, & Co., Mountain View, CA), and data were analyzed using C6 software (ver. 1.0.264.21). For NAC treatment, cells were pretreated with 1 mM NAC for 2 h, after which, the cells were collected and exposed to HCY or acrolein as described above.

### Measurement of lactate dehydrogenase release

The presence of lactate dehydrogenase (LDH), regarded as a marker of cellular injury, was measured in cell-culture supernatant. H9c2 cells (1.25 × 10^4^ cells/well) were cultured overnight in 96-well plates. After incubation, the cells were exposed to HCY (1.25–40 μM) and acrolein (3–100 μM) at 37 °C in 5% CO_2_. After 8 h incubation, the amount of LDH released in the medium of each sample was assayed, according to the manufacturer’s instructions, using LDH Cytotoxicity Detection Kits (Cat. #MK401; Takara, Shiga, Japan). LDH catalyzes the dehydrogenation of lactic acid and produces pyruvic acid and NADH. This NADH reduces the tetrazolium salt with the catalyst of diaphorase and forms red formazan that absorbs 490 nm wavelength radiation. Consequently, LDH activity can be evaluated by measuring 490 nm absorbance. Briefly, after 8 h incubation, 100 μL samples of medium were placed in each well of new 96-well plates. Then, 100 μL of reaction mix was added to each well. After 30 min incubation, using a microplate reader (Infinite M200, Tecan, Mannedorf, Switzerland), 492 nm absorbance was measured, along with background absorbance at 600 nm, and the data analyzed using i-Control Ver. 1.0 software (Tecan).

### Measurement of acrolein concentration in cell culture by high performance liquid chromatography

H9c2 cells were exposed to 10 μM HCY for 2 h. After 2 h exposure, cell culture supernatants were collected and acrolein was measured using a colorimetric method based on the specific reaction of acrolein with m-aminophenol in the presence of hydroxylamine [13]. Using the method of Bohnenstengel et al. [11, 14], the amount of acrolein was determined by high performance liquid chromatography (HPLC).

H9c2 cells were also exposed to 100 μM acrolein for 4 h with and without 1 mM NAC. After 4 h exposure, cell culture supernatants were collected and the amount of acrolein was determined by the sample extraction and HPLC methods described in the preceding paragraph.

### Detection of apoptosis by evaluation of caspase-3 and caspase-7 activity

To detect apoptosis, caspase-3 and caspase-7 activity was evaluated. As described above in “Analysis of cell viability”, H9c2 cells were plated at 2.5 × 10^4^ cells/mL density in 24-well plates and exposed to 30 μM HCY or 30 or 100 μM acrolein for 4 h. After different treatments, the supernatants were removed and cells were washed with PBS. Then, the PBS was replaced with 500 μL of phenol-red-free DMEM supplemented with 10% FBS. Finally, a drop of CellEvent Caspase 3/7 Green ReadyProbes reagent (Cat. # R37111; Life Technologies) was added to each well and incubated for 30 min at 37 °C in 5% CO_2_, and the cells were observed using 100× magnification fluorescence microscopy (Axio Observer Z1). To stain live cell nuclei, a drop of NucBlue Live Cell Stain ReadyProbes reagent (Cat. #R37605; Life Technologies), which includes Hoechst 33342 fluorescence dye, was added to each well for 20 min at room temperature. We conducted this procedure for the products of three experiments. Live cells were consequently stained blue and apoptotic cells green.

### Measurement of cellular glutathione content

Amounts of intracellular glutathione (GSH) were evaluated using NWLSS Glutathione Assay kits (Northwest Life Science Specialties, LLC, Vancouver, WA). H9c2 cells were plated at 2.5 × 10^4^ cells/mL density in 25 cm^2^ tissue culture flasks, and grown, at 37 °C in 5% CO_2_, to 90% confluence. After incubation, the cells were exposed to 30 μM of acrolein, with and without, NAC at 37 °C in 5% CO_2_. For experiments with NAC, the cells were pre-treated as described above in “Analysis of cell viability”, and then incubated for 2 h at 37 °C in 5% CO_2_. Untreated cell samples were used for control. After treatment, cells were collected and assayed for GSH content as described in our previous report [11].

### Evaluation of ALDH activity

ALDH activity was analyzed using PicoProbe Aldehyde Dehydrogenase Activity Fluorometric Assay kits (BioVision Inc., Milpitas, CA). Briefly, in 25 cm^2^ tissue culture flasks, H9c2 cells were plated at 2.5 × 10^4^ cells/mL density and grown, at 37 °C in 5% CO_2_, to 90% confluence. After incubation, the cells were exposed, at 37 °C in 5% CO_2_, to 10 μM HCY or 30 μM acrolein, with and without 1 mM NAC. For experiments with NAC, the cells were pre-treated as described above in “Analysis of cell viability”, and then exposed, at 37 °C in 5% CO_2_, for 4 h to HCY or acrolein with NAC. Untreated cells were used for control. After treatment, cells were collected and twice washed with ice-cold PBS. The cells were then re-suspended with assay buffer and homogenized by sonication. The lysates were centrifuged at 12,000 rpm for 5 min at 4 °C and the supernatant was collected. This supernatant was diluted 20-fold with assay buffer. Thus treated, the supernatant was collected and, according to the manufacturer’s instructions, assayed for GSH content.

### Statistical analysis

Data are presented as mean ± standard deviation (SD). Statistical analysis was performed using StatView version 5.0 for Windows (SAS, Institute Inc., Cary, NC). Treatment effects were established by nonparametric Wilcoxon tests. A probability value of less than 0.05 was considered to be statistically significant.

## Results

### Myocardial cytotoxicity induced by HCY and acrolein

**Fig. 2:**
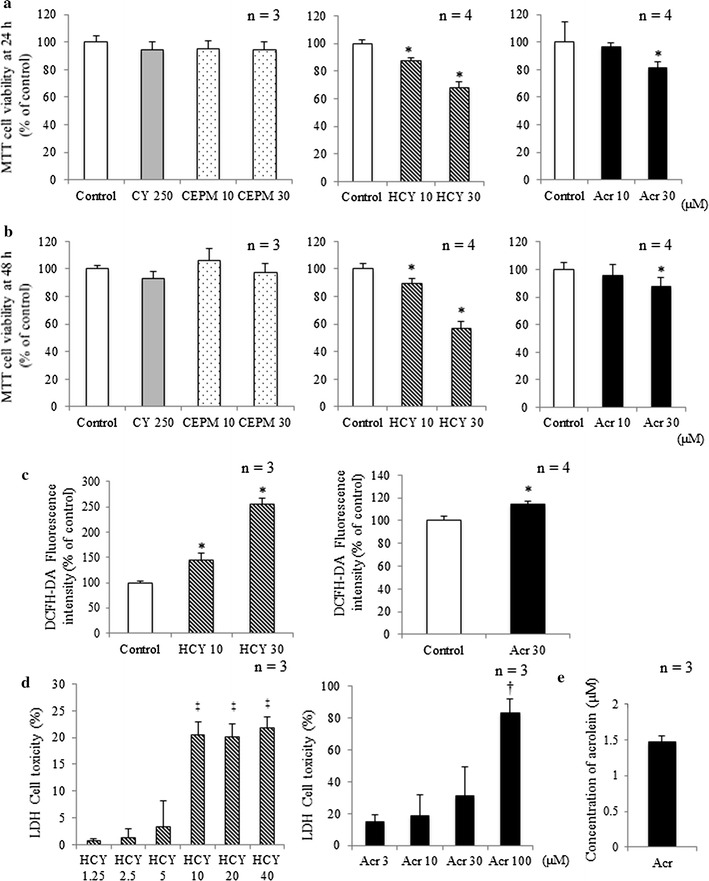
Myocardial cytotoxicity induced by HCY or acrolein. H9c2 cell viability after **a** 24-h and **b** 48-h exposure to CY alone and CY metabolites was assessed by MTT assay (mean + SD from 3 to 4 independent experiments). **c** Fluorescence intensities, corresponding to levels of H_2_O_2_, in control samples or cells exposed to 10 or 30 μM HCY and 30 μM acrolein for 15 min (mean + SD from 3 to 4 independent experiments). Fluorescence intensity is shown in arbitrary units. **d** LDH release from H9c2 cells exposed to HCY and acrolein for 8 h (mean + SD from three independent experiments). **e** Concentration of acrolein in cell culture medium after exposure to 10 μM HCY. H9c2 cells were exposed for 2 h to HCY. Changing concentrations of acrolein in culture media were evaluated using HPLC. *p < 0.05 compared with control. ^†^ *p* < 0.05 compared with 3 μM acrolein. ^‡^ *p* < 0.05 compared with 1.25 μM HCY

Assay results for MTT showed that exposures to CY, CEPM or 10 μM acrolein did not induce cytotoxicity. Myocardial cytotoxicity did ensue, however, after 24 and 48 h exposure to 10 or 30 μM HCY and 30 μM acrolein (Fig. 2a, b; *p* < 0.05). Intracellular levels of ROS were evaluated from fluorescence intensity measured by flow cytometry: in 10 or 30 μM HCY and 30 μM acrolein samples, intracellular levels of ROS were higher than in control samples (Fig. 2c; *p* < 0.05). LDH release assay results showed greater presence in 10, 20 or 40 μM HCY (*p* < 0.05) and 100 μM acrolein (*p* < 0.05) samples than in low-dose HCY (3 μM) or acrolein (1.25 μM; Fig. 2d) samples. The average concentration of acrolein in cell culture media after 2 h exposure to 10 μM HCY was 1.5 ± 0.07 μM (Fig. 2e).

### Inhibition of CY metabolites-induced cell cytotoxicity by NAC

**Fig. 3:**
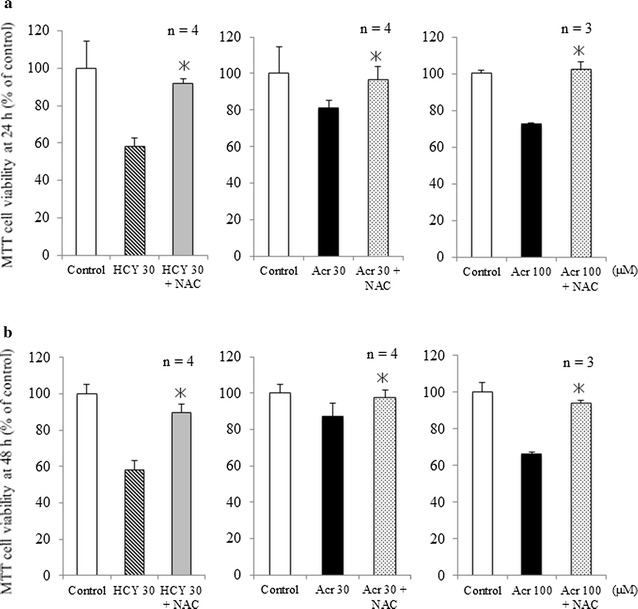
Inhibition by NAC of cell cytotoxicity induced by CY metabolites. **a** Effect of NAC on cytotoxicity of CY metabolites in H9c2 cells after 24-h exposure (mean + SD from 3 to 4 independent experiments conducted in duplicate). **b** Effect of NAC on cytotoxicity of CY metabolites in H9c2 cells after 48-h exposure (mean + SD from 3 to 4 independent experiments conducted in duplicate). **c** Effect of NAC on ROS generated by CY metabolites, as shown by fluorescence intensity of DCFH-DA in cells exposed for 15 min to 30 μM HCY or 30 μM acrolein plus NAC. Fluorescence intensity is shown in arbitrary units (mean + SD from three independent experiments). *p < 0.05 compared with samples without NAC

MTT assay results of 24- and 48-h samples showed that NAC inhibited cytotoxicity induced by CY metabolites (HCY, acrolein) compared with control samples (Fig. 3a, b; *p* < 0.05).

### Optical and fluorescence images of H9c2 cells exposed to CY metabolites and CY metabolites plus NAC

**Fig. 4:**
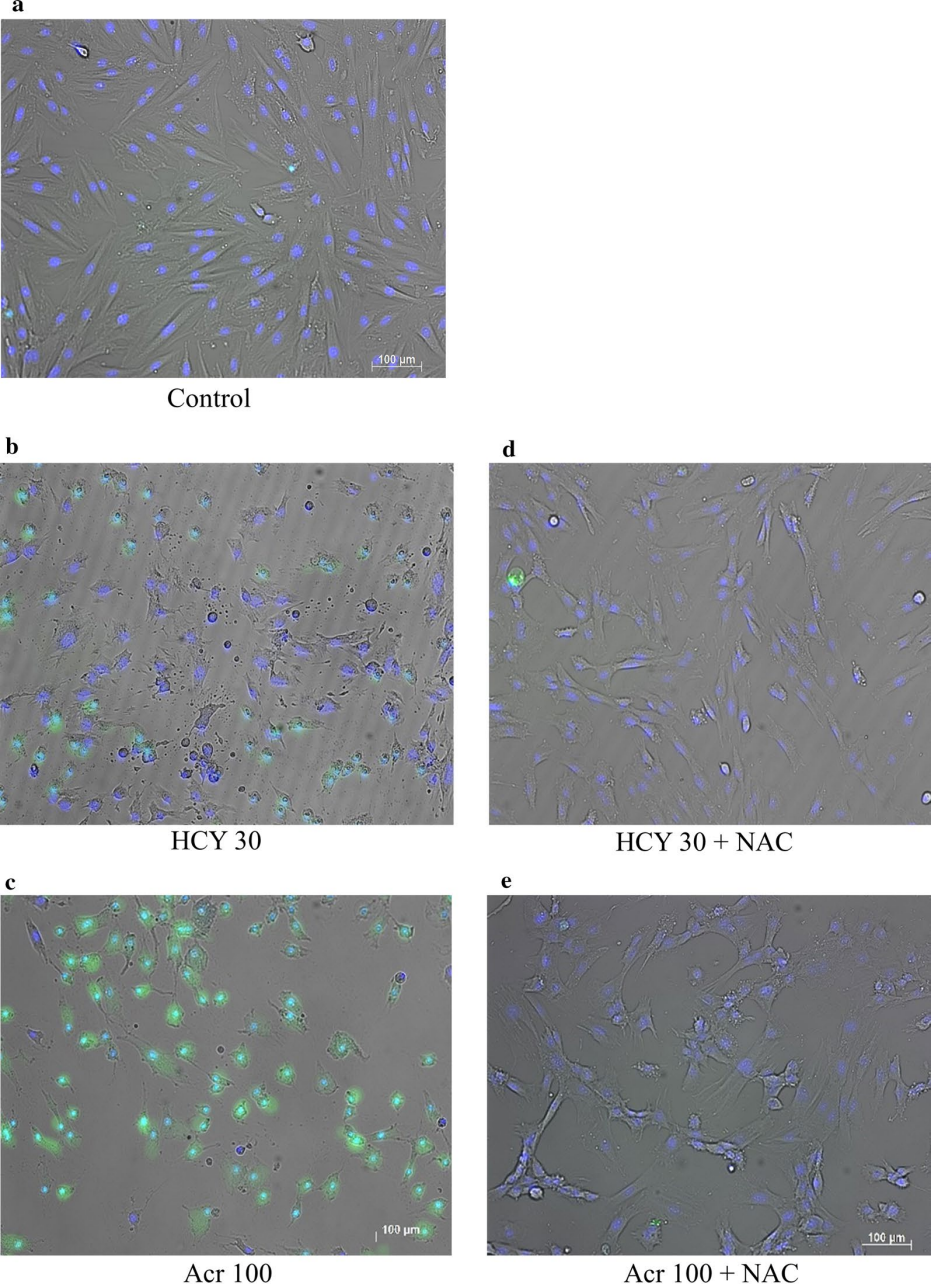
Optical and fluorescence images of H9c2 cells exposed, in presence or absence of NAC, to CY metabolites. **a**–**e** Optical images of samples after 24 h exposure: living cell nuclei stained by Hoechst 33342 are blue. Induction of apoptosis in H9c2 cells by CY metabolites in presence or absence of NAC: living cell nuclei stained by Hoechst 33342 are blue; apoptotic cells stained by FITC-conjugated probes are green. **a** Control (unexposed H9c2 cells); **b** H9c2 cells exposed to 30 μM HCY; **c** H9c2 cells exposed to 100 μM acrolein. **d** H9c2 cells exposed to 30 μM HCY with NAC; **e** H9c2 cells exposed to 100 μM acrolein with NAC. Magnification, ×100; *bar* 200 μm

Live-cell imaging of samples, in 24-well plates, with cell density of 2.5 × 10^4^ cells/mL, also revealed acute cytotoxicity in the presence of CY metabolites and inhibition of this by NAC (Fig. 4a–e). Samples with CY metabolites without NAC, showed shrunken or irregularly shaped cells; but in samples with NAC, cells had a very similar appearance to those in control samples. The protective effect of NAC on H9c2 cells was further verified using Hoechst 33342 staining and fluorescent assays of caspase-3/7 activity. In Fig. 4d, e, cells treated with NAC and challenged by CY metabolites have the same kind of round nuclei and homogeneous blue fluorescence intensity as normal cells. As Fig. 4c shows, caspase-3 and caspase-7 activity was greater in 100 μM acrolein samples; such activity was suppressed in samples pre-treated for 2 h with 1 mM NAC (Fig. 4e).

### NAC inhibited ROS generation associated with CY metabolites

**Fig. 5:**
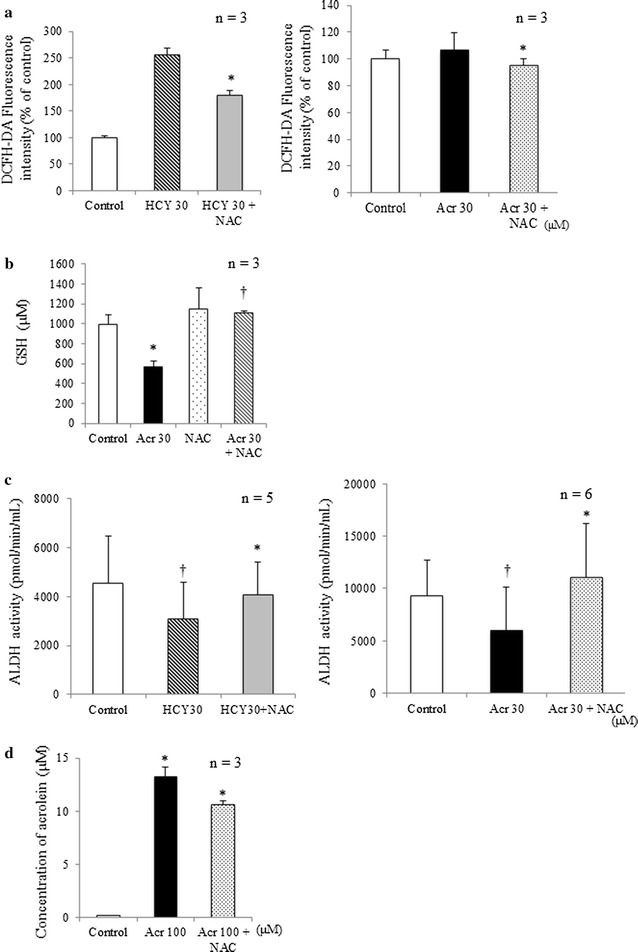
Various effects of NAC on H9C2 cells after exposure to CY metabolites. **a** ROS generation in H9c2 cells after exposure to HCY or acrolein in presence or absence of NAC. In H9c2 cell samples exposed to 30 μM HCY and 30 μM acrolein for 15 min (mean + SD from three independent experiments), the presence of NAC prevented increased ROS generation. For control, unexposed H9c2 cells were used. *p < 0.05 compared with samples without NAC. **b** Reduced glutathione levels in H9c2 cells after exposure to acrolein in presence or absence of NAC. Effects of NAC on glutathione levels in H9c2 cells exposed to acrolein for 2 h (mean + SD from three independent experiments). For control, unexposed H9c2 cells were used. *p < 0.05 compared with control samples; ^†^ *p* < 0.05 compared with acrolein exposure. **c** ALDH activity in H9c2 cells after exposure to HCY or acrolein with and without NAC. Effects of NAC on ALDH activity in H9c2 cells exposed to 30 μM HCY and 30 μM acrolein for 4 h (mean + SD from 5 to 6 independent experiments). For control, unexposed H9c2 cells were used. Compared with control samples, ALDH activity decreased in 30 μM HCY and 30 μM acrolein. Presence of NAC prevented decrease in ALDH activity. ^†^ *p* < 0.05 compared with control group; *p < 0.05 compared with samples without NAC. (D) Acrolein concentration in H9c2 cells after exposure to 100 μM acrolein in presence or absence of NAC. H9c2 cells were exposed for 4 h to acrolein (mean + SD from three independent experiments). The changes of acrolein in culture media was measured using HPLC. The concentration of acrolein was decreased in the presence of NAC. *p < 0.05 compared with 100 μM acrolein without NAC

After exposure to 30 μM HCY and 30 μM acrolein, myocytes in samples that had been pretreated with NAC showed statistically significantly less ROS generation (Fig. 5a).

### NAC inhibited GSH depletion associated with acrolein

Exposure to 30 μM of acrolein was statistically significantly (p < 0.05) associated with decreased cardiomyocyte GSH. No such decrease was apparent in myocyte samples pre-treated with NAC and then exposed to acrolein (Fig. 5b).

### NAC inhibited lessening of ALDH activity by CY metabolites

Compared with control samples, exposure to 30 μM HCY or 30 μM acrolein was statistically significantly associated with less ALDH activity. The same kind of samples pretreated with NAC, showed statistically significantly more ALDH activity than those that were not pretreated (Fig. 5c).

### Acquisition of acrolein by NAC

The average concentration of acrolein in cell culture media after 4 h exposure to 100 μM acrolein was 13.2 ± 0.94 μM. With NAC, however, after exposure to the same concentration for the same time, the average acrolein concentration was 10.6 ± 0.37 μM (Fig. 5d). This confirms that NAC captured acrolein in the samples.

## Discussion

Since the mechanism of fatal cardiotoxicity that may attend high-dose CY has not yet been elucidated, and no definitive risk factors have yet been identified, we investigated possible cardiotoxic mechanisms. This follows our previous report on CY cardiotoxicity using CY metabolized by rat liver homogenate, S9 (CYS9) in vitro. Results indicated that CY itself is not cardiotoxic, rather, the harm is caused by CY metabolites [11]. It remained unclear, however, specifically how CY metabolites are involved in cardiotoxicity. Our findings suggested that acrolein plays a major role in CY cardiotoxicity. We designed the current study to investigate, by exposing H9c2 cells to CY metabolites, which metabolites are implicated in cardiotoxicity.

The concentrations of the three CY metabolites used in this study were determined based on results from pharmacokinetic studies of high-dose cyclophosphamide in patients and from in vitro studies [11]. While CEPM did not exhibit myocardial cytotoxicity, HCY at concentrations of 10 and at 30 μM, and acrolein at 30 μM were clearly cytotoxic at 24 and 48 h (Fig. 2a, b). We further tested whether HCY was converted to acrolein in the cell culture and found, after 2 h exposure to 10 μM HCY, that the concentration of acrolein in cell culture medium was about 1.5 μM (Fig. 2e). There was an ongoing conversion of HCY to acrolein in the culture medium. HCY itself is probably cardiotoxic, but it causes more damage if converted to acrolein. Besides being a CY metabolite, acrolein is also a ubiquitous environmental pollutant: as a reactive aldehyde, it is of great concern to public health. In recent years, acrolein has been implicated in cardiac diseases [15, 16], and it is a known cause of CY-induced hemorrhagic cystitis [17]. Another basic study has suggested that acrolein may be involved in hepatic disorders, including veno-occlusive disease [18].

We previously showed that NAC inhibits CY-induced cardiotoxicity [11], and our present results from MTT assay, Hoechst33342 staining and fluorescent assays of caspase-3/7 activity indicate that NAC also inhibits cardiotoxicity induced both by HCY and by acrolein (Figs. 3, 4). In either case, pre-treatment with NAC inhibited the production of intracellular ROS (Fig. 5a). This finding is consistent with other reports attributing CY-induced cardiotoxicity to an increase in free oxygen radicals and induced apoptosis [19–21]. GSH is a potent intracellular anti-oxidant and it has been reported that intracellular depletion of GSH enhances cytotoxicity and apoptosis [22]. In line with our previous findings [11], results in Fig. 5b indicate that while acrolein statistically significantly decreased intracellular GSH from the level in control samples, the presence of NAC was associated with increased intracellular GSH. This NAC-increased GSH level may play an important role in the mechanism protecting H9c2 cells from the effects of CY metabolites.

In the CY metabolic pathway, AldoCY is metabolized to CEPM in the presence of ALDH [4]; if ALDH activity decreases, metabolism to CEPM is inhibited, and the level of acrolein increases. We previously found, compared with control samples, that pre-treatment with NAC statistically significantly increases CEPM concentration and decreases acrolein concentration, and concluded that NAC inhibits CYS9-induced cardiotoxicity [11]. Ren et al. have also reported that acrolein inhibits ALDH1 activity [23]. Here, we further found (Fig. 5c) statistically significantly less ALDH activity after exposure to HCY or acrolein, but that this decrease was counteracted by NAC. For H9c2 cells, this NAC-increased ALDH activity may also play an important role in the protective mechanisms.

Yoshida et al. have reported that NAC is a powerful scavenger of acrolein [12]. Indeed, we found that pre-treatment with NAC statistically significantly decreased acrolein concentration compared to control samples (Fig. 5d). In cell samples challenged by CY metabolites, directly scavenging acrolein, NAC promisingly maintained the level of ALDH activity. Cardiac myocytes may defend themselves against the cytotoxicity of high-dose CY by expressing ALDH, which plays a similar defensive role in cancer stem cells [24] and in hematopoietic stem cells [25].

## Conclusions

Cardiotoxicity induced by CY is dose-dependently related to the presence of HCY and acrolein. Although high-dose CY therapy is an essential treatment for the eradication of tumor cells, some patients succumb to cardiotoxicity. Cardiotoxicity induced by high-dose CY is associated with ROS generation, GSH depletion, and lessened ALDH activity, and our findings suggest that NAC is likely to protect against CY-induced cardiotoxicity by counteracting these effects. NAC is widely used as a mucolytic agent [26] and as an antidote for hepatotoxicity cause by acetaminophen overdoses [27, 28]. Furthermore, since for preventing oral mucositis in HSCT settings, NAC is already being used from day one in high-dose chemotherapy that may involve the use of CY, effective clinical application of NAC to prevent high-dose CY cardiotoxicity is feasible [29]. In clinical practice, by measuring acrolein in blood plasma and treating accordingly with NAC, CY-induced cardiotoxicity may be prevented.

## Abbreviations

ALDH: aldehyde dehydrogenase
CEPM: carboxyethylphosphoramide mustard
CY: cyclophosphamide
DCFH-DA: dichlorofluorescin diacetate
FITC: fluorescein isothiocyanate
GSH: glutathione
H9c2: rat cardiac myocardial cell line
HCY: 4-hydroxy-cyclophosphamide
HPLC: high performance liquid chromatography
MTT: 3-(4,5-dimethyl-2-thiazolyl)-2,5-diphenyl-2*H*-tetrazolium bromide
NAC: *N*-acetylcysteine
PBS: phosphate-buffered saline
ROS: reactive oxygen species
